# Adverse childhood experiences, resilience, and cannabis use in early motherhood

**DOI:** 10.3389/fpsyt.2025.1621161

**Published:** 2025-08-07

**Authors:** Alysa Roland, Elizabeth Charron, Karina M. Shreffler

**Affiliations:** ^1^ Fran and Earl Ziegler College of Nursing, University of Oklahoma Health Sciences, Oklahoma, OK, United States; ^2^ Hudson College of Public Health, University of Oklahoma Health Sciences, Tulsa Schusterman Center, Tulsa, OK, United States

**Keywords:** adverse childhood experiences, cannabis, resilience, postpartum, maternal

## Abstract

**Background:**

Exposure to Adverse Childhood Experiences (ACEs) is associated with increased risk of substance use in women, including cannabis use during pregnancy. Less is known, however, about how resilience factors moderate the association of ACEs on cannabis use in early motherhood.

**Methods:**

We used survey data from 126 predominately low-income and diverse mothers enrolled in a longitudinal study in the South Central U.S. Multiple logistic regression models evaluated associations between ACEs and cannabis use through three years postpartum, stratified by resilience scores (median split). Adjusted models controlled for sociodemographic factors, postnatal depression, and prenatal substance use. Average predicted probabilities were estimated from fully adjusted models.

**Results:**

Among individuals with high resilience, each unit increase in ACEs was associated with significantly higher odds of cannabis use in early motherhood (adjusted OR = 1.38; 95% CI: 1.07–1.78). No significant association was observed among those with low resilience (adjusted OR = 1.02; 95% CI: 0.77–1.34). In the high resilience group, the average predicted probability of cannabis use increased from 8.5% at 0 ACEs to 62.9% at 10 ACEs; in the low resilience group, average predicted probabilities of cannabis use was high (~36%) regardless of ACE score.

**Conclusions:**

The findings suggest that although ACEs are a social determinant of cannabis use in early motherhood, resilience may be protective, particularly among those with low and moderate ACE exposure. However, its protective effect diminishes with higher ACE exposure.

## 1 Introduction

Adverse childhood experiences (ACEs) continue to be a public health concern, with nearly 70% of the U.S. population experiencing one or more ACEs in 2022, and 23% experiencing at least four or more (1). Strong evidence exists for the link between ACEs and poorer health and wellbeing during adulthood, including negative impacts on physical and mental health (2, 3), disruption in interpersonal relations (4), and increased participation in health-harming behaviors like substance use (5). Inequities persist for women and minoritized people who are more likely to experience severe ACEs than other groups (6). Recent evidence suggests that resilience may protect against the adverse mental health effects of ACEs during the perinatal period (7, 8), but less is known about its role to moderate the behavioral consequences of ACEs, which could have important implications for health behavior policy and practice.

Cannabis use is common and increasing among women of childbearing ages, in large part due to recent legislation for recreational and/or medical use in the majority of U.S. states (9). Between 1992 and 2022, cannabis consumption in the U.S. increased 15-fold, and daily or near-daily cannabis use is now more common than daily or near-daily alcohol use (17.7 million daily or near-daily cannabis users as compared to 14.7 million daily or near-daily alcohol users) (10). Cannabis use in pregnancy is associated with numerous adverse maternal and child health outcomes, including 50% greater risk for perinatal mortality, 40% higher risk for preterm birth, and twice the risk of developmental delay at 12 months of age (11). Given the widespread prevalence of cannabis use during pregnancy (16.0% nationally before 2020) and recent dramatic increases in use (12), cannabis associated developmental delays are the second most common cause of developmental disabilities, after alcohol (11).

Cannabis use during early motherhood is also not without risk. When lactating women use cannabis, Delta-9-tetrahydrocannabinol (THC) and other cannabis metabolites can accumulate in breastmilk for up to 6 days after last use, exposing breastfed infants (13, 14). Additionally, cannabis use alters breastmilk composition including macronutrient makeup and immunoglobin levels (14, 15), which has unknown impacts on infant development. Substance use in the home also poses risks for secondhand exposure and accidental ingestion (16), intergenerational transfer of ACEs (17, 18), and increased negative parenting behaviors (19). For example, cannabis use may impair working memory (20), which is an essential function when caring for infants and young children (21).

Although most women who use cannabis before pregnancy discontinue use during pregnancy, as many as two-thirds reinitiate use during the postpartum period (22, 23). This period can be a particularly high-risk time for return to cannabis use due to increased stress, ongoing mental health symptoms, and prior health conditions (24, 25). Postpartum women commonly report using cannabis to relax, reduce stress, improve mood, and manage both mental and physical health symptoms (23, 26, 27). While the postpartum period is often defined as ending between 6 weeks and 1 year after delivery (28, 29), the motives that drive cannabis use often persist beyond this timeframe (30) and underscores the need to examine potential protective factors across the early motherhood period.

Resilience generally refers to the ability to bounce back or recover from threats following adversity (31) and is associated with favorable maternal and child health outcomes (32, 33). Resilience can be enhanced through positive relationships and strong connections with others (34) and is a modifiable target for intervention (35). Recent evidence suggests that resilience buffers adverse maternal mental health consequences for those with a history of ACEs. Young-Wolff and colleagues (8) found that in pregnancy, higher ACE scores are associated with higher depression and anxiety among those with low levels of resilience, and Armans and colleagues (7) found that high levels of resilience are protective against pregnancy stress for those with low to moderate ACE scores.

While prior research has established a link between ACEs and cannabis use in pregnancy (36), less is known about the relationship between ACEs and cannabis use in early motherhood. Specifically, no studies to date have examined the moderating role of resilience in this relationship, which could identify important targets for interventions to support maternal health and reduce substance use after pregnancy. This study draws on the Health Belief Model (37) to conceptualize how resilience may influence health-risk behaviors like cannabis use, given experiences of early adversity. Although the effects of ACEs may increase an individual’s risk for stress-related coping, resilience may increase behavioral control, reduce reliance on maladaptive coping, and alter beliefs about risks versus benefits of use. The aim of this study was to examine the association between ACEs and cannabis use in early motherhood and to explore whether resilience moderates this relationship. We anticipated that higher ACEs would be associated with cannabis use, and that higher resilience would buffer the association between ACEs and cannabis use.

## 2 Methods

### 2.1 Data source and study population

The current study is a secondary analysis of survey data collected as part of a longitudinal study conducted in the South Central U.S. that examined the effects of maternal stressors on maternal and child health and well-being outcomes. The study population was recruited from a clinical population of racially diverse pregnant and postpartum women seeking prenatal care at one of two university-affiliated women’s health clinics. Potential participants were assessed for eligibility at their first prenatal care visit. Individuals were eligible to participate if they were less than 16 weeks’ gestation at enrollment, planning to continue their pregnancy, and planning to be a primary caretaker of the offspring. Those unable to speak and read English or under the age of 15 or over the age of 45 were ineligible for participation. Participants gave informed consent, and those under 18 gave informed assent and had parental consent for participation. Study enrollment occurred between 2017 and 2018 and participants were followed through December 2020. Enrolled participants completed online self-report questionnaires across nine timepoints during pregnancy, the postpartum period, and early motherhood through two years after delivery. Cannabis legalization for medical reasons occurred during the postpartum phase of the study. For the current analysis, we included only participants with complete data for the outcome of interest (i.e., cannabis use in early motherhood) (*n*=126). The study was approved by the authors’ university Institutional Review Board.

### 2.2 Variables

#### 2.2.1 Exposure

The *Adverse Childhood Experiences* (ACEs) Questionnaire (2) assesses 10 categories of adverse experiences before the age of 18, including abuse (physical, emotional, sexual), neglect (physical, emotional), and household dysfunction (e.g., parental separation/divorce, household substance abuse, mental illness, domestic violence, incarceration). Each “Yes” response is scored as 1 point, yielding a total score ranging from 0 to 10, with higher scores indicating greater exposure to ACEs. In this study, the ACE score was treated as a continuous variable.​ For descriptive purposes only, we also categorized ACE scores into mild (0–1), moderate (2–3), and severe (≥4) exposure groups based on established cutpoints, with a score of 4 or higher being commonly used to define severe exposure (2, 7, 38).

#### 2.2.2 Outcome

The outcome of interest was *cannabis use during early motherhood*, defined as any self-reported use as measured with the question, “Do you currently use marijuana?” at three separate timepoints: approximately six months postpartum (assessment 6), approximately 15 months postpartum (assessment 8), and approximately 22 months postpartum (assessment 9). We created a binary variable indicating cannabis use if participants endorsed use at any of the three timepoints (0 = no, 1 = yes); those who responded “no” at all timepoints were categorized as not using cannabis in early motherhood.

#### 2.2.3 Potential moderator

*Resilience* was assessed using the Brief Resilience Scale (BRS), a 6-item validated instrument designed to measure the ability to recover from stress (39). Participants responded to each item using a five-point Likert scale ranging from 1 (strongly disagree) to 5 (strongly agree). Items include: “I tend to bounce back quickly after hard times,” “I have a hard time making it through stressful events,” “It does not take me long to recover from a stressful event,” “It is hard for me to snap back when something bad happens,” “I usually come through difficult times with little trouble,” and “I tend to take a long time to get over setbacks in my life.” Negatively worded items (“I have a hard time making it through stressful events,” “It is hard for me to snap back when something bad happens,” “I tend to take a long time to get over setbacks in my life”) were reverse coded before calculating scores. The final score was calculated by summing responses to the six items, giving a total score ranging from 6-30, with higher scores indicating greater resilience.

#### 2.2.4 Covariates

Covariates included *age*, *years of education*, *race/ethnicity* (White, Black, Hispanic, American Indian/Alaska Native), *union status* (married/cohabiting vs. not cohabiting/single/divorced/widowed/separated), *parity* (number of previous births), *postnatal depression*, and *prenatal substance use*, including alcohol, tobacco, opioids, and cannabis. Participants were asked, “Since getting pregnant, how often having you been … using [alcohol/tobacco/opioids/marijuana]?” Never responses were coded as “0”, and any use was coded as a “1” for each type of substance use. Depression was assessed using the 20-item Center for Epidemiologic Studies Depression Scale (CES-D), a widely used and validated measure of depressive symptoms (40). Scores range from 0 to 60, with a score of 16 or higher indicative of clinical depression.

### 2.3 Statistical analysis

Descriptive statistics were used to compare characteristics of the sample and ACEs scores stratified by cannabis use during early motherhood. Group comparisons were conducted using Student’s t tests, chi-squared tests, or Mann-Whitney U tests, as appropriate. We evaluated dose-response relationships between the number of adverse childhood experiences (ACEs) and cannabis use using unadjusted and adjusted logistic regression models, with results presented as odds ratios (ORs) and 95% confidence intervals (CIs). Adjusted models controlled for age, educational attainment, race/ethnicity, union status, parity, postnatal depression, and prenatal use of alcohol, tobacco, opioids, and cannabis. All covariates, except race/ethnicity, were modeled as continuous or dichotomous variables due to sample size constraints. To assess whether the association between ACEs and cannabis use varied by resilience, we included resilience as a continuous variable and specified an interaction term for continuous ACEs × continuous resilience in the fully adjusted model. Given the statistically significant interaction, we present regression models stratified by resilience level to illustrate the modifying effect of resilience on the association between ACEs and cannabis use. To facilitate interpretation, resilience was dichotomized at the sample median (BRS score = 22), which is a commonly used data-driven approach in the absence of a validated cut-point (41). We report stratified ORs and 95% CIs comparing individuals with high (above median) versus low (at or below median) resilience. As a final step, we estimated and plotted average predicted probabilities of cannabis use across ACE scores, stratified by resilience level, using marginal effects from the adjusted models.

Missing data >5% were present for several covariates, including education (10.3%), depression (19.8%), and prenatal substance use (6.4%). We examined patterns of missing data and found no significant differences in characteristics between participants with and without missing values, suggesting data were likely missing completely at random. To address missingness, we used multiple imputation via the Markov Chain Monte Carlo (MCMC) method under the assumption of multivariate normality (42). Multivariable logistic regression models were conducted across five imputed datasets, and results were pooled using standard Rubin’s rules (43). As a sensitivity analysis, we performed a complete case analysis data to ensure that imputation did not affect our results. We additionally conducted a sensitivity analysis without adjusting for prenatal cannabis use to assess whether results changed when this covariate was excluded. All tests were two-sided, and statistical significance was defined as *p* < 0.05. Data management and analyses were conducted using SAS version 9.4 (SAS Institute, Cary, NC) and Stata version 17 (StataCorp LLC, College Station, TX).

## 3 Results

Among the 126 participants, 17.5% (*n* = 31) reported cannabis use during early motherhood (**Table 1**). Cannabis use was reported by 9 participants (7.1%) at assessment six, 18 (14.3%) at assessment eight, and 18 (14.3%) at assessment nine, with 10 participants (7.8%) reporting use at multiple timepoints and 4 (3.2%) at all three timepoints. Self-reported prenatal alcohol use was significantly more common among individuals who reported cannabis use compared to those who did not (20.0% vs. 6.8%; *p* = 0.04). Similarly, self-reported prenatal cannabis use was more frequent among those who used cannabis during early motherhood (25.8% vs. 6.3%; *p* = 0.002). The mean ACEs score was significantly higher among individuals who reported cannabis use compared to non-users (mean [SD]: 4.2 [3.4] vs. 2.9 [2.8]; *p* = 0.045). When ACEs were categorized, a greater proportion of cannabis users reported severe ACEs (scores 4–10) compared to non-users (54.8% vs. 31.6%; *p* = 0.04). No statistically significant differences were observed between groups with respect to age, education, race/ethnicity, union status, parity, postnatal depression, prenatal use of tobacco or opioids, or resilience.

**Table 1:** Characteristics of individuals reporting cannabis use and no cannabis use in early motherhood.

Characteristics	Cannabis use (*n*=31) *n* (%)	No cannabis use (*n*=95) *n* (%)	*P-*value
Sociodemographic			
Age, years (mean [SD])	26.7 (5.9)	25.6 (5.4)	0.32
Education, years (mean [SD])	12.5 (1.5)	12.5 (1.9)	0.88
Race/Ethnicity			0.24
White	13 (41.9)	37 (39.8)	
Black	11 (35.5)	24 (25.8)	
Hispanic	1 (3.2)	16 (17.2)	
AI/AN	6 (19.4)	16 (17.2)	
Union	20 (64.5)	57 (60)	0.65
Parity (mean [SD])	1.58 (1.86)	1.22 (1.33)	0.24
Mental health and substance use			
Postnatal depression^a^	11 (45.8)	29 (37.7)	0.48
Prenatal alcohol use	6 (20.0)	6 (6.8)	0.04^b^
Prenatal tobacco use	9 (30.0)	20 (22.5)	0.41
Prenatal opioid use	5 (16.7)	11 (11.6)	0.47
Prenatal cannabis use	8 (25.8)	6 (6.3)	0.002^b^
Maternal ACEs			
ACEs (mean [SD])	4.2 (3.4)	2.9 (2.8)	0.045^b^
ACEs, categories			
Mild ACEs (0-1)	12 (38.7)	46 (48.4)	0.04^b^
Moderate ACES (2-3)	2 (6.5)	19 (20.0)	
Severe ACEs (4-10)	17 (54.8)	30 (31.6)	
Resilience score			
Resilience (mean [SD])	21.5 (3.2)	21.6 (3.8)	0.94

SD, standard deviation; AI/AN, American Indian and Alaskan Native; ACE, adverse childhood experiences. All data listed as n (%) unless otherwise noted. Categorical ACEs for descriptive purposes only.

a Depression was measured using the CES-D scale and dichotomized as having depression (cutoff > 16) and not having depression (< 16).

b Significant p-values (*p* < 0.05).

**Figure 1:**
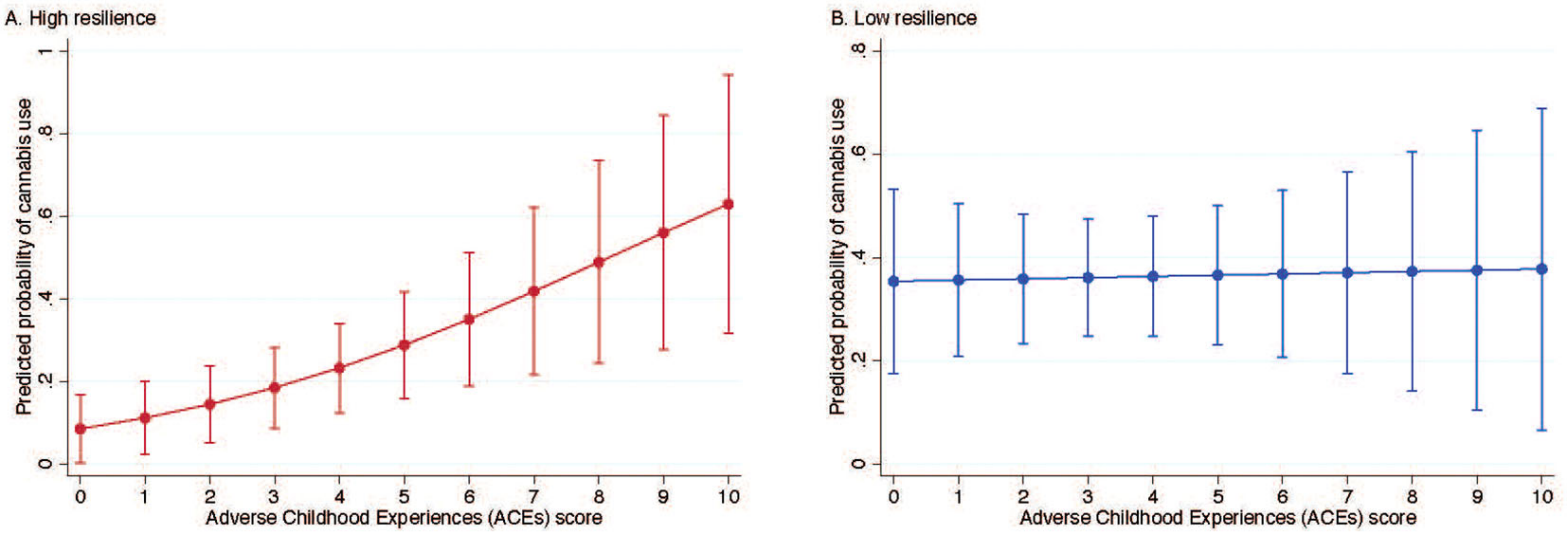
Predicted probability of cannabis use by ACE score, stratified by resilience level.

In the unadjusted and adjusted models, each unit increase in ACEs was significantly associated with higher odds of cannabis use in early motherhood (unadjusted OR, 1.15; 95% CI, 1.00-1.31; adjusted OR, 1.20; 95% CI, 1.01-1.43) (data not shown). In the fully adjusted model, the interaction between ACEs score and resilience was statistically significant (*p* = 0.04), indicating that the effect of ACEs score on cannabis use in early motherhood varied by level of resilience (supplemental material, **Supplementary Table S1**). Among individuals with high resilience, each unit increase in ACEs score was associated with higher odds of cannabis use in early motherhood in both the unadjusted (OR = 1.26, 95% CI: 1.04–1.52) and adjusted models (OR = 1.38, 95% CI: 1.07–1.78) (**Table 2**). No significant association between ACEs score and cannabis use was observed among those with low resilience in either the unadjusted (OR = 1.01, 95% CI: 0.81–1.24) or adjusted models (OR = 1.02, 95% CI: 0.77–1.34). Sensitivity analyses produced similar results to the main findings (supplemental material, **Supplementary Tables S2**, **S3**). Among those with high resilience, the probability of cannabis use increased from 8.5% (95% CI: 1.9–16.8) at 0 ACEs to 62.9% (95% CI: 31.7–94.1) at 10 ACEs (**Figure 1A**). Among those with low resilience, predicted probabilities were relatively stable, ranging from 35.4% (95% CI: 17.5–53.2) at 0 ACEs to 37.8% (95% CI: 6.6–68.9) at 10 ACEs (**Figure 1B**).

**Table 2:** Associations between adverse childhood experiences and cannabis use in early motherhood, stratified by resilience level.

	Cannabis use (*n*=31)	No cannabis use (*n*=95)
High resilience		
Mean (SD)	24.5 (2.0)	24.3 (2.3)
Unadjusted OR (95% CI)	1.26 (1.04-1.52)	Reference
Adjusted OR (95% CI)a	1.38 (1.07-1.78)	Reference
Low resilience		
Mean (SD)	19.1 (1.5)	18.4 (2.5)
Unadjusted OR (95% CI)	1.01 (0.81-1.24)	Reference
Adjusted OR (95% CI)^a^	1.02 (0.77-1.34)	Reference

SD, standard deviation; OR, odds ratio; CI, confidence interval.

No significant *p*-values (*p* < 0.05).

a Models adjusted for age, education, race-ethnicity, union status, parity, postnatal depression, and prenatal substance use (alcohol, tobacco, opioids, and cannabis).

## 4 Discussion

The aim of this study was to examine the association between ACEs and cannabis use in early motherhood and to explore whether resilience moderates this relationship. ACEs have been associated with prenatal substance use including alcohol (44), cannabinoids (45), cannabis (36, 46) and other substance use (45–47). Kendall-Tackett et al. (48) also found that ACEs were associated with frequency of cannabis use among pregnant women but when the number of health problems were accounted for, ACEs were no longer a significant predictor of cannabis use behavior. In our sample, higher ACEs were associated with an increased likelihood of cannabis use during early motherhood among those with high resilience, but no association was observed among those with low resilience. These findings suggest that resilience may influence how early life adversity relates to cannabis use behaviors in the postpartum period.

Together, our findings suggest that resilience influences the extent to which adversity affects cannabis use behavior. Specifically, individuals with low resilience may have an elevated risk of cannabis use that is not influenced by ACE exposure. That is, individuals with low resilience may engage in higher rates of cannabis use overall, so the marginal effect of increasing ACEs on the probability of cannabis use is small because the behavior is already elevated. Prior research has shown that when positive coping mechanisms are unavailable, individuals are likely to use substances to manage distress (49). In individuals with low resilience, mental health conditions and maladaptive coping strategies may be more common (8, 50), contributing to sustained or elevated rates of cannabis use, especially in early motherhood when stress levels are high (51). Coping is also a commonly reported motivation for cannabis use (52, 53) and may contribute to worsening stress responses, creating a cycle of repeated substance use and distress (54), even in the absence of additional trauma exposure.

On the other hand, although higher resilience has been found to mitigate negative outcomes in various contexts (7, 8, 32, 33), we found that resilience did not appear to protect against cannabis use among individuals with high ACE exposure, especially among those who reported experiencing 6–10 ACEs. Among individuals with high resilience, there may be limits to its protective effects under conditions of high or chronic adversity, such as exposure to numerous ACEs. Prior research has shown that exposure to multiple ACEs has a detrimental impact on healthy development (2–5) and adaptation (55, 56) to the degree that resilience may not protect against the influence from adversity. Individuals may engage in high-risk behaviors like substance use to regulate or alleviate symptoms related to adversity experiences including developmental effects (17), lower self-esteem (57) and depression (58).

Although the findings seem contradictory, other researchers have also found that resilience is less effective at high levels of ACEs. For example, Armans et al. (7) found that high resilience moderated the association between ACEs and pregnancy-specific stress at low and moderate levels of ACEs but not severe ACEs. A similar pattern has been described in studies examining the effects of resilience on the development of personality disorders and engagement in health risk behaviors in young adults exposed to childhood adversity, where resilience was less protective at higher levels of adversity (56). In contrast, Young-Wolf (8) reported that high resilience was protective against mental health conditions at high levels of ACEs. Together with extant literature, our findings indicate that the cumulative impact of multiple experiences of childhood adversity may be profound enough such that resilience is inadequate to mitigate some outcomes–particularly for behavioral outcomes such as cannabis use.

Providers caring for women and children should remain aware of the relationship between low resilience, adversity, and cannabis use and consider providing brief intervention to increase positive coping behaviors among mothers (59). Although high individual resilience was not protective in our sample for those with high ACE scores, providers can work with families to increase family resilience, which has been shown to improve outcomes among families impacted by ACEs (60).

### 4.1 Strength and limitations

Our findings should be interpreted in light of several strengths and limitations. A major strength of the study is its prospective, longitudinal design, which allowed for the collection of data across pregnancy and into early motherhood. We also used validated measures for key constructs, including ACEs, resilience, and depressive symptoms, which strengthens the reliability of the findings. First, however, all measures for the present study, including cannabis use and resilience, were collected through participant self-report. We recognize that including more objective measures, in particular for cannabis use, would have strengthened the validity of the assessments. However, prior research suggests that self-reported substance use is often underreported (61), and such underreporting would likely influence prevalence estimates rather than bias the direction of observed associations. Second, self-reported measures capture static responses to constructs that may fluctuate over time, such as resilience or patterns of cannabis use, limiting the ability to capture dynamic changes. Third, some of the covariates in our model (e.g., age, education, tobacco use) were not significant predictors of cannabis use despite their well-supported documentation in prior research (62–64). These findings may demonstrate that cannabis use during early motherhood is becoming more common across demographic groups, or that our study was underpowered to detect small effect sizes. Fourth, although we adjusted for a range of conceptually important covariates, the possibility of residual confounding from unmeasured variables cannot be ruled out. Finally, the modest sample size and focus on a specific regional clinical population may limit the generalizability of the findings to broader or more diverse populations. Future research can expand this work with representative data and by examining additional factors that buffer the effects of ACEs on health behaviors and can be modified through intervention.

## 5 Conclusion

This study documents the moderating role of resilience on the association between ACEs and cannabis use in early motherhood. Among those with lower levels of resilience, cannabis use was high regardless of ACEs. Among those with higher levels of resilience, cannabis use was low among those with low and moderate levels of childhood adversity. Among those with high ACE scores (particularly among those with more than 6 adverse childhood experiences), cannabis use was high. This suggests that resilience influences the extent to which adversity affects cannabis use behavior, and while it is protective against cannabis use at lower levels of adversity, it does not appear to reduce cannabis use among those with high levels of adversity.

## Data Availability

The raw data supporting the conclusions of this article will be made available by the authors, without undue reservation.
