# Supplementary material for: Adverse childhood experiences, resilience, and cannabis use in early motherhood
Source: Front Psychiatry. 2025 Aug 7;16:1621161. doi: 10.3389/fpsyt.2025.1621161 (PMC12368428; doi:10.3389/fpsyt.2025.1621161)
Supplement: Supplementary file 1 [file Table1.docx]

**SUPPLEMENTAL MATERIAL**

**Table S1.** Interaction between continuous ACEs and continuous resilience in relation to postpartum cannabis use, from fully-adjusted regression model

|  |  |  |  |
| --- | --- | --- | --- |
|  | β Estimate | SE | *P-*value |
| ACEs score | -1.16 | 0.69 | 0.09 |
| Resilience | -0.27 | 0.18 | 0.13 |
| ACEs × Resilience | 0.07 | 0.03 | 0.04 |

Abbreviations: β = beta; SE = standard error; ACEs = adverse childhood experiences.

^a^ Models adjusted for age, education, race-ethnicity, union status, parity, postnatal depression, and prenatal substance use (alcohol, tobacco, opioids, and cannabis).

**Table S2.** Associations between adverse childhood experiences and cannabis use in early motherhood, stratified by resilience level, using complete case analysis

|  |  |  |
| --- | --- | --- |
|  | Cannabis use  (n=31) | No cannabis use  (n=95) |
| High resilience |  |  |
| Adjusted OR (95% CI)^a^ | 1.89 (1.12-3.20) | Reference |
| Low resilience |  |  |
| Adjusted OR (95% CI)^a^ | 1.15 (0.83-1.60) | Reference |

Abbreviations: OR = odds ratio; CI = confidence interval

^a^ Models adjusted for age, education, race-ethnicity, union status, parity, postnatal depression, and prenatal substance use (alcohol, tobacco, opioids, and cannabis).

**Table S3.** Associations between adverse childhood experiences and cannabis use in early motherhood, stratified by resilience level, without prenatal cannabis use as a covariate

|  |  |  |
| --- | --- | --- |
|  | Cannabis use  (n=31) | No cannabis use  (n=95) |
| High resilience |  |  |
| Adjusted OR (95% CI)^a^ | 1.38 (1.07-1.77) | Reference |
| Low resilience |  |  |
| Adjusted OR (95% CI)^a^ | 0.98 (0.75-1.26) | Reference |

Abbreviations: OR = odds ratio; CI = confidence interval

^a^ Models adjusted for age, education, race-ethnicity, union status, parity, postnatal depression, and prenatal substance use (alcohol, tobacco, and opioids).
